# From task validation to workflow assurance in health technology assessment

**DOI:** 10.1017/S0266462326104012

**Published:** 2026-07-21

**Authors:** Gaurav Agarwal, Sharmila Chaudhuri

**Affiliations:** 1 Department of Economics, Centre for Distance and Online Education, Galgotias University, India; 2 Department of Physiotherapy, School of Allied Health Sciences, Galgotias University, India

We read with great interest the ongoing *Dialogue on Artificial Intelligence in Health Technology Assessment (HTA)*, particularly the article by Fleurence and Chhatwal on implementing generative artificial intelligence (GenAI) into HTA practice (1) and the subsequent letter by Yu et al., which extends these principles to bedside AI and clinical data governance (2). Together, these contributions converge on an important principle: GenAI should be validated according to well-defined, bounded tasks rather than viewed as a technology that is uniformly “ready” or “not ready” for HTA.

We fully agree with this perspective and wish to extend it one step further. Current validation approaches primarily evaluate GenAI within individual tasks, such as literature screening, data extraction, or report drafting. HTA recommendations, however, are generated through interconnected workflows in which the output of one stage becomes the input to the next. As Yu et al. note, translating AI capability into clinical practice requires human oversight and appropriate governance (2). We suggest that it also requires attention to how AI-assisted outputs interact across successive stages of the HTA process.

This distinction has practical implications. For example, omission of a clinically important study during literature screening may influence subsequent evidence synthesis, alter inputs to an economic model, and ultimately affect reimbursement recommendations, even when downstream tasks are themselves performed accurately. Strong performance for individual components therefore does not necessarily guarantee the reliability of the complete HTA workflow.

Future evaluations could usefully complement task-specific benchmarking with workflow-level validation, examining how outputs propagate across chained tasks and whether the overall assessment remains robust. Such an approach would provide a practical extension of the framework proposed by Fleurence and Chhatwal by identifying where human oversight and accountability should be exercised throughout the HTA pathway, rather than only within individual tasks (1).

The Dialogue has convincingly established that GenAI should be validated task by task. We suggest that the next methodological step is to evaluate how these validated tasks perform collectively as an integrated workflow, thereby strengthening trust, accountability, and the reliability of AI-enabled HTA.

## Data Availability

Data sharing is not applicable to this article as no new data were created or analyzed in this study.
